# Predictive value of lipoprotein(a) for left atrial thrombus or spontaneous echo contrast in non-valvular atrial fibrillation patients with low CHA_2_DS_2_-VASc scores: a cross-sectional study

**DOI:** 10.1186/s12944-023-01990-1

**Published:** 2024-01-22

**Authors:** Kamila Kamili, Tingting Zheng, Chaodi Luo, Xuan Wang, Gang Tian

**Affiliations:** Department of Cardiovascular Medicine, First Affiliated Hospital of Xi’an Jiaotong University, 277 Yanta West Rd, Xi’an, Shaanxi 710061 People’s Republic of China

**Keywords:** Left atrial spontaneous echo contrast, Left atrial thrombus, CHA2DS2-VASc score, Atrial fibrillation, Lipoprotein(a)

## Abstract

**Objective:**

Current guidelines are debated when it comes to starting anticoagulant therapy in patients with non-valvular atrial fibrillation (NVAF) and low CHA_2_DS_2_-VASc scores (1–2 in women and 0–1 in men). However, these individuals still have a high likelihood of developing left atrial thrombus/spontaneous echo contrast (LAT/SEC) and experiencing subsequent thromboembolism. Recent research has demonstrated that lipoprotein(a) [Lp(a)] may increase the risk of thrombosis, but the relationship between Lp(a) and LAT/SEC in NVAF patients is not clearly established. Therefore, this study sought to evaluate the predictive ability of Lp(a) for LAT/SEC among NVAF patients with low CHA_2_DS_2_-VASc scores.

**Methods:**

NVAF patients with available transesophageal echocardiography (TEE) data were evaluated. Based on the TEE results, the subjects were classified into non-LAT/SEC and LAT/SEC groups. The risk factors for LAT/SEC were examined using binary logistic regression analyses and were validated by using 1:1 propensity score matching (PSM). Subsequently, novel predictive models for LAT/SEC were developed by integrating the CHA_2_DS_2_-VASc score with the identified factors, and the accuracy of these models was tested using receiver operating characteristic (ROC) analysis.

**Results:**

In total, 481 NVAF patients were enrolled. The LAT/SEC group displayed higher Lp(a) concentrations. It was found that enlarged left atrial diameter (LAD), high concentrations of Lp(a), and a history of coronary heart disease (CHD) were independent predictors of LAT/SEC. Lp(a) and LAD still had predictive values for LAT/SEC after adjusting for PSM. In both the highest quartile groups of Lp(a) (>266 mg/L) and LAD (>39.5 mm), the occurrence of LAT/SEC was higher than that in the corresponding lowest quartile. By incorporating Lp(a) and the LAD, the predictive value of the CHA_2_DS_2_-VASc score for LAT/SEC was significantly improved.

**Conclusion:**

Elevated Lp(a) and enlarged LAD were independent risk factors for LAT/SEC among NVAF patients with low CHA_2_DS_2_-VASc scores. The prediction accuracy of the CHA_2_DS_2_-VASc score for LAT/SEC was significantly improved by the addition of Lp(a) and LAD. When evaluating the stroke risk in patients with NVAF, Lp(a) and LAD should be taken into account together with the CHA_2_DS_2_-VASc score.

**Trial registration:**

Retrospectively registered.

## Introduction

Among individuals with non-valvular atrial fibrillation (NVAF), the occurrence of left atrial thrombus (LAT) is directly linked to stroke. Anticoagulant therapy can eliminate atrial thrombosis and reduce the risk of subsequent embolism [1]. Moreover, the presence of left atrial spontaneous echo contrast (SEC), indicating a prothrombotic condition, serves as an indication for initiating anticoagulation therapy [2].

The CHA_2_DS_2_-VASc score is an updated version of the CHADS_2_ score, which is commonly employed for stroke risk stratification and provides guidance for clinical anticoagulation therapy [3]. Current guidelines recommend anticoagulation medication for patients with high CHA_2_DS_2_-VASc scores; however, the necessity of anticoagulation therapy for those with low scores (women: 1-2 points; men: 0-1 point) is debatable. Nevertheless, these patients still face a significant risk of LAT/SEC and subsequent thromboembolism [4–6].

Lipoprotein (a) [Lp(a)] is one of the frequently found components of blood lipids [7]. Lp(a) has been considered a potential marker for thrombosis [8]. Previous studies have also shown a considerable increase in the prevalence of LAT in those with high concentrations of Lp(a) [9]. Nevertheless, the correlation between Lp(a) and LAT/SEC in those with low scores is not fully understood. Thus, this study sought to evaluate the ability of Lp(a) to predict LAT/SEC in individuals with low CHA_2_DS_2_-VASc scores, to identify patients who may have undetected thrombosis based on the current scoring system, and to provide timely oral anticoagulant treatment to enhance outcomes.

## Methods

### Study population

**Fig. 1:**
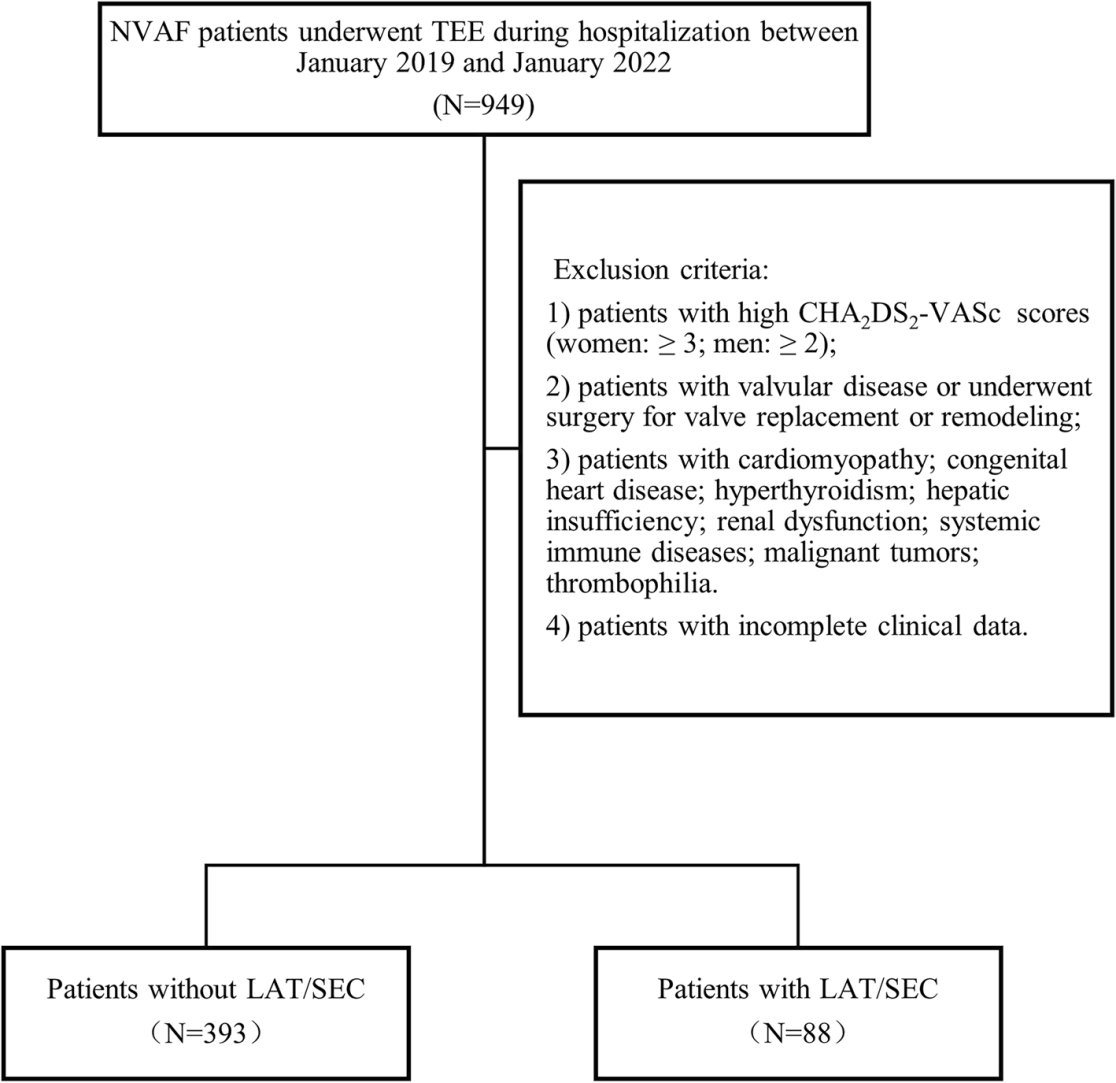
Flowchart with inclusion and exclusion criteria for the study. NVAF: non-valvular atrial fibrillation; TEE: transesophageal echocardiography; LAT/SEC: left atrial thrombus/spontaneous echo contrast

Through a systematic review of medical records, this research gathered data from 949 patients with NVAF, all of whom underwent transesophageal echocardiography (TEE) and transthoracic echocardiography (TTE) prior to left atrial appendage closure (LAAC) or radio-frequency catheter ablation (RFCA) during hospitalization between January 2019 and January 2022 in the Department of Cardiology, First Affiliated Hospital of Xi'an Jiaotong University. The following patients were disqualified based on the exclusion criteria: 1) patients with high CHA_2_DS_2_-VASc scores (women: ≥ 3 points; men: ≥ 2 points); 2) patients with valvular disease or those who have undergone surgery for valve replacement or remodeling; 3) patients with cardiomyopathy; 4) patients with congenital heart disease; 5) patients with other systemic diseases, such as hyperthyroidism, severe hepatic and renal insufficiency, systemic immune diseases, malignant tumors, and thrombophilia; 6) patients with incomplete clinical data. Eventually, 481 patients were included. All the relevant information, including demographic parameters, echocardiography results, and laboratory examination results, was gathered from the electronic medical records system. Figure 1 shows the flowchart with inclusion and exclusion criteria for the study.

### CHA_2_DS_2_-VASc score

Based on the clinical information provided, the CHA_2_DS_2_-VASc score was recalculated. This scoring system assigns 1 point for each risk variable, including diabetes mellitus, vascular disease, hypertension, and congestive heart failure. Specifically, individuals aged 65–74 years received 1 point, while those aged 75 years or older received 2 points. A transient ischemic attack (TIA) or stroke received 2 points. Females were assigned an additional point. Females with scores of 1-2 and males with scores of 0-1 were classified as having low CHA_2_DS_2_-VASc scores [3].

### Echocardiographic examination

All patients underwent TEE to exclude LAT or SEC prior to receiving RFCA or LAAC. The diagnostic criteria for LAT are well-defined as independent, mobile, circular, oval, or irregular shapes with uniform density that differ from the surrounding myocardial tissue density and can be detected in multiple sections of the left atrial cavity [10]. The diagnostic criteria for SEC include the observation of swirling, smoke-like, or prethrombotic states within the left atrium (LA), which must be distinguished from illusions caused by changes in near-field and high-gain artifacts [11]. TTE was conducted to collect data on cardiac chamber size and ventricular wall motion to evaluate cardiac structures and functions. Echocardiography was performed by two professional ultrasound physicians. One physician was responsible for performing the operation and making the diagnosis, while the other physician reviewed the results. Both physicians were unaware of the other clinical data of the patient before the examination.

### Statistical analyses

Continuous variables were displayed as medians (interquartile ranges) or means ± standard deviations ($\overline{{\text{X}} }$ ± SDs), depending on whether they followed a normal distribution and were compared employing Student's t tests or corrected t tests. Variables with a categorical nature were displayed as frequencies (n) and percentages (%). For comparison, Fisher's exact test or the chi-square test was performed. The risk variables for LAT/SEC were identified using multivariate logistic regression. The baseline imbalance was corrected using propensity score matching (PSM) with a caliper value of 0.01 for optimal matching. The predictive capacity of Lp(a) and other risk variables was evaluated through the receiver operating characteristic (ROC) analysis. For statistical significance, a *P-*value of less than 0.05 was required.

## Results

481 NVAF patients with an average age of 56.81±8.88 years were included. Based on the TEE results, the subjects were classified into the LAT/SEC group (*n* = 88) and the non-LAT/SEC group (*n* = 393). LAT was found in 60 patients, including 33 patients with combined SEC; SEC alone was found in 28 patients. The incidences of LAT and SEC accounted for 12.47% and 11.43%, respectively, of the total population. 6.1% of patients in the non-LAT group underwent LAAC, and 32.1% underwent RFCA. As shown in Table 1, coronary heart disease (CHD) (9.1% vs. 2.3%) was more prevalent in the LAT/SEC group (9.1% vs. 2.3%), as was non-paroxysmal atrial fibrillation (48.9% vs. 29.5%). A higher diastolic blood pressure (83.5 (76–90) mmHg vs. 79 (73–87) mmHg, *P* < 0.05) and a higher Lp (a) level [281.50 (178.25–394.75) mg/L vs. 146.00 (86.50–229.00) mg/L, *P* < 0.01] were found in the LAT/SEC group. Additionally, activated partial thromboplastin time (APTT), NT-proBNP, and prothrombin time (PT) were also higher in this group. Furthermore, the LAT/SEC group exhibited an enlarged left atrial diameter (LAD) and a decreased left ventricular ejection fraction (LVEF). No statistical differences were observed in terms of CHA_2_DS_2_-VASc score, age, gender, smoking and alcohol consumption history, systolic blood pressure, pulse, hypertension, prior heart failure, stroke, or previous use of oral anticoagulants or other medications.

**Table 1:** Baseline characteristics of NVAF patients with/without LAT/SEC

	**Total**(***n*=481)**	**Non-LAT/SEC group(*n*=393)**	**LAT/SEC group(*n*=88)**	***P*-value**
CHA_2_DS_2_-VASc score	1(0-1)	1(0-1)	1(0-1)	0.657
Paroxysmal AF, n	322(66.9)	277(70.5)	45(51.1)	<0.001*
Age, years	56.81±8.88	56.55±9.23	57.97±7.01	0.315
Male, n	317(65.9)	262(66.7)	55(62.5)	0.456
Smoke, n	179(37.2)	154(39.2)	25(28.4)	0.059
Alcohol consumption, n	71(14.8)	58(14.8)	13(14.8)	0.997
BMI, kg/m^2^	25.19±3.03	25.20±3.03	25.18±3.05	0.951
SBP, mmHg	121.40±18.09	121.42±18.63	121.30±15.57	0.955
DBP, mmHg	80(73-88)	79(73-87)	83.5(76-90)	0.008*
HR, bpm	75(66-85)	74(66-84)	77(67-88.75)	0.181
Hypertension, n	153(31.8)	120(30.5)	33(37.5)	0.205
Diabetes, n	19(4.0)	19(4.8)	0(0)	0.035*
CHD, n	17(3.5)	9(2.3)	8(9.1)	0.002*
Stroke/TIA, n	5(1.0)	5(1.3)	0(0)	0.287
Heart failure, n	10(2.1)	6(1.5)	4(4.5)	0.073
Anticoagulants, n	153(31.8)	118(30.0)	35(39.8)	0.076
Antiplatelet agents, n	81(16.8)	63(16.0)	18(20.5)	0.316
Statins, n	100(20.8)	82(20.9)	18(20.5)	0.932
RFCA, n	102(21.2)	102(26.1)	0	-
LAAC, n	19(4.0)	19(4.9)	0	-
RBC, ×10^12^/L	4.68(4.38-5.01)	4.68±0.53	4.75±0.50	0.239
MCV, fl	93.60(90.60-96.50)	93.80(90.80-96.90)	92.55(89.80-94.75)	0.014*
MCH, pg	31.00(30.10-32.00)	31.20(30.10-32.10)	30.75(30.03-31.50)	0.025*
PLT, ×10^9^/L	193.82±56.70	194.93±57.40	189.32±54.19	0.410
WBC, ×10^9^/L	5.70(4.71-6.86)	5.65(4.70-6.74)	5.89(4.87-7.35)	0.195
ALP, U/L	71.00(58.50-84.00)	70(58-83)	78(63.25-93.75)	0.004*
γ-GT, U/L	23.00(16.00-37.00)	23(15-35.50)	27.50(18.00-40.75)	0.058
BUN, mmol/L	6.18(5.28-7.39)	6.13(5.23-7.26)	6.58(5.51-7.63)	0.104
Scr, umol/L	0.87(0.73-1.02)	62.91±16.80	59.80±18.60	0.124
TC, mmol/L	3.69(3.09-4.26)	3.70(3.16-4.29)	3.53(2.92-4.14)	0.115
TG, mmol/L	1.11(0.82-1.58)	1.12(0.82-1.60)	1.05(0.81-1.52)	0.552
HDL-C, mmol/L	0.97(0.85-1.14)	0.98(0.86-1.16)	0.94(0.81-1.07)	0.067
LDL-C, mmol/L	2.14(1.61-2.68)	2.22±0.78	2.15±0.85	0.467
ApoA, g/L	1.11(1.02-1.24)	1.12(1.02-1.25)	1.08(1.02-1.17)	0.052
ApoB, g/L	0.72±0.21	0.72±0.21	0.70±0.23	0.414
Lp(a), mg/L	164.00(91.50-266.00)	146.00(86.50-229.00)	281.50(178.25-394.75)	<0.001*
PT, s	12.70(11.30-14.10)	12.50(11.30-13.80)	13.45(11.63-15.60)	0.003*
APTT, s	30.60(26.80-36.70)	30.20(26.73-36.30)	34.10(27.45-39.20)	0.035*
FIB, g/L	2.63(2.29-3.04)	2.63(2.29-3.04)	2.61(2.24-2.99)	0.573
D-Dimer, mg/L	0.23(0.13-0.39)	0.24(0.13-0.39)	0.19(0.10-0.37)	0.219
NT-proBNP, pg/mL	348.3(102.50-770.70)	286.20(87.82-651.70)	703.90(302.80-1367.00)	<0.001*
LAD, mm	35.00(31.00-39.50)	34.0(31.0-39.0)	39.0(35.0-43.0)	<0.001*
IVSA, mm	8.00(7.00-9.00)	8.0(7.0-9.0)	8.0(7.0-9.0)	0.027*
MPAD, mm	22.00(20.00-24.00)	22.0(20.0-24.0)	23.0(21.0-24.0)	0.252
LVEF, %	66.00(62.00-70.00)	67.0(62.0-70.0)	65.0(60.25-69.0)	0.038*

^*^P <0.05

*AF* Atrial fibrillation, *ALP* Alkaline phosphatase, *ApoA* Apolipoprotein A, *ApoB* Apolipoprotein B, *APTT* activated partial thromboplastin time, *BMI* Body Mass Index, *BUN* Blood urea nitrogen, *CHD* Coronary heart disease, *DBP* Diastolic Blood Pressure, *FIB* Fibrinogen, *HDL-C* High-density lipoprotein cholesterol, *HR* Heart rate, *IVSA* Interventricular septal amplitude, *LAD* Left atrial diameter, *LAAC* Left atrial appendage closure, *LAT/SEC* Left atrial thrombosis/spontaneous echo contrast, *LDL-C* Low-density lipoprotein cholesterol, *Lp(a)* Lipoprotein(a), *LVEF* Left ventricular ejection fraction, *MCH* Mean corpuscular hemoglobin, *MCV* Mean corpuscular volume, *MPAD* Main pulmonary artery, *PLT* Platelet, *PT* Prothrombin time, *RBC* Red blood cells, *RFCA* Radio-frequency catheter ablation, *SBP* Systolic Blood Pressure, *Scr* Serum creatinine, *TC* Total cholesterol, *TG* Triglyceride, *TIA* Transient Ischemic Attacks, *WBC* White blood cells, *γ-GT* γ-glutamyl transferase

### Risk factors for LAT/SEC

By incorporating variables that have been demonstrated to be associated with LAT formation in previous research (age, gender [12], non-paroxysmal atrial fibrillation, LVEF [13], fibrinogen [14], and D-dimer [15]), as well as risk factors identified through univariate regression analyses in the present study, multivariate regression analyses were performed and the results demonstrated that LAD, CHD history, and Lp(a) level were all predictors of LAT/SEC (Table 2).

**Table 2:** Univariable and multivariate logistic regression for risk factors of LAT/SEC

	Univariable			Multivariate		
	OR	95%CI	*P* -value	OR	95%CI	*P* -value
Paroxysmal AF	0.438	0.274-0.702	0.001*	1.342	0.755-2.388	0.316
DBP	1.026	1.005-1.048	0.014*	1.021	0.998-1.044	0.077
CHD	4.267	1.598-11.395	0.004*	4.559	1.530-13.587	0.006*
Heart failure	3.071	0.848-11.124	0.087			
Anticoagulants	1.539	0.954-2.483	0.077			
MCV	0.989	0.975-1.003	0.132			
MCH	0.963	0.923-1.005	0.082			
ALP	1.002	0.998-1.007	0.335			
γ-GT	0.999	0.996-1.003	0.715			
Lp(a)	1.003	1.002-1.004	<0.001*	1.003	1.002-1.005	<0.001*
PT	1.111	1.046-1.181	0.001*	1.028	0.985-1.072	0.200
APTT	1.001	0.992-1.009	0.909			
NT-proBNP	1.001	1.000-1.001	<0.001*	1.000	1.000-1.001	0.104
LAD	1.119	1.074-1.166	<0.001*	1.088	1.032-1.146	0.002*
IVSA	0.850	0.742-0.973	0.019*	0.943	0.777-1.145	0.555
LEVF	0.977	0.953-1.002	0.076			

^*^P <0.05

*AF* Atrial fibrillation, *ALP* Alkaline phosphatase, *APTT* Activated partial thromboplastin time, *CHD* Coronary heart disease, *CI* Confidence interval, *DBP* Diastolic Blood Pressure, *IVSA* Interventricular septal amplitude, *LAD* Left atrial diameter, *Lp(a)* Lipoprotein (a), *LVEF* Left ventricular ejection fraction, *MCH* Mean corpuscular hemoglobin, *MCV* Mean corpuscular volume, *OR* Odds ratio, *PT* Prothrombin time, *γ-GT* γ-glutamyl transferase

**Fig. 2:**
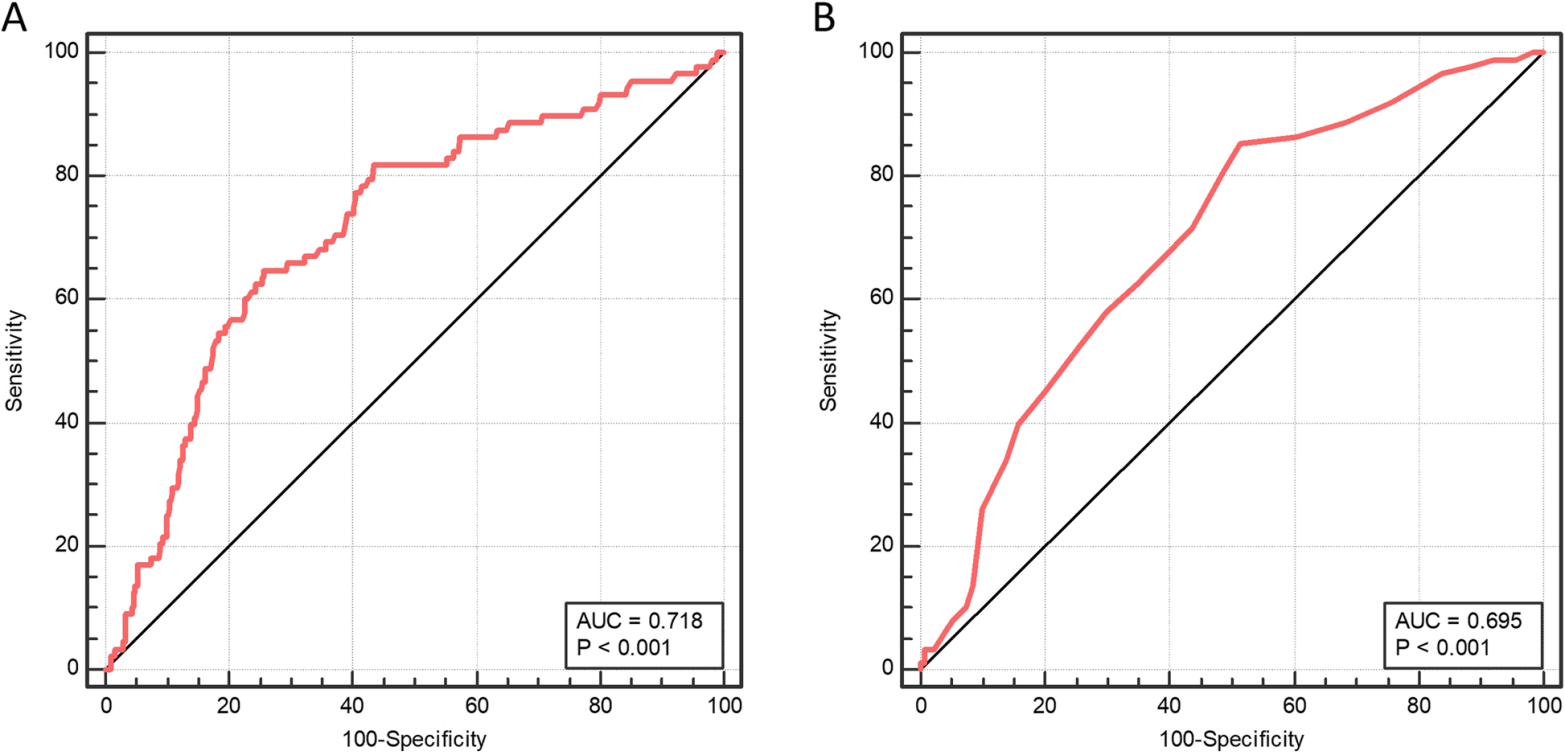
Receiver operating characteristic (ROC) curves for the prediction of left atrial thrombus/spontaneous echo contrast (LAT/SEC) of lipoprotein (a) [A] and left atrial diameter (LAD) [B]. AUC: area under the curve

Subjects were subsequently separated into four groups according to the quartile of LAD and Lp(a) levels. The Lp(a) level was favorably linked with LAT/SEC formation according to further multivariate logistic regression analysis (Table 3). Specifically, individuals in the highest quartile group exhibited a greater incidence of LAT/SEC compared to the first quartile group. Even after making adjustments for gender and age in Model I, as well as additional confounding factors including history of CHD, diastolic blood pressure, PT, D-dimer, fibrinogen, NT-proBNP, LAD, LVEF, and interventricular septal amplitude (IVSA) in Model II, these associations remained consistent. Similarly, regardless of whether adjustments were made for confounding factors, there was a positive correlation between an increased prevalence of LAT/SEC and left atrial enlargement. As illustrated in Fig. 2, the ROC curves showed the prediction capacity of Lp(a) and LAD for LAT/SEC. The AUC for the LAD was 0.695 (95% confidence interval [CI] 0.637-0.753, *P* < 0.01), while that for Lp(a) was 0.718 (95% CI 0.658-0.778, *P* < 0.01). According to these findings, both LAD and Lp(a) were risk factors for LAT/SEC.

**Table 3:** Multivariate logistic analysis of LAT/SEC with plasma Lp(a) level and LAD

	Crude		Model I		Model II	
	OR (95% CI)	*P*	OR (95% CI)	*P*	OR (95% CI)	*P*
Lp(a), mg/L	1.003 (1.002-1.004)	<0.001*	1.003 (1.002-1.004)	<0.001*	1.003 (1.002-1.005)	<0.001*
Q1	ref.	ref.	ref.	ref.	ref.	ref.
Q2	0.991 (0.379-2.590)	0.098	0.970 (0.370-2.541)	0.950	0.667 (0.235-1.889)	0.446
Q3	2.769 (1.217-6.297)	0.015*	2.663 (1.168-6.075)	0.020*	2.150 (0.886-5.218)	0.091
Q4	8.222 (3.802-17.780)	<0.001*	8.263 (3.804-17.950)	<0.001*	8.495 (3.692-19.548)	<0.001*
LAD, mm	1.072 (1.017-1.130)	0.009*	1.118 (1.072-1.165)	<0.001*	1.088 (1.032-1.146)	0.002*
Q1	ref.	ref.	ref.	ref.	ref.	ref.
Q2	1.918 (0.825-4.456)	0.130	1.932 (0.826-4.518)	0.129	1.485 (0.594-3.714)	0.398
Q3	3.100 (1.407-6.830)	0.005*	3.097(1.389-6.908)	0.006*	2.517 (1.023-6.192)	0.045*
Q4	6.200 (2.935-13.096)	<0.001*	6.174 (2.882-13.226)	<0.001*	3.548 (1.002-9.025)	0.008*

^*^P<0.05

*LAD* Left atrial diameter, *LAT/SEC* Left atrial thrombosis/spontaneous echo contrast, *Lp(a)* Lipoprotein (a), *OR* Odds ratio, *CI* Confidence interval

Model I adjusted for gender and age. Mode II adjusted for gender, age, non-paroxysmal atrial fibrillation, smoke, history of coronary heart disease, diastolic blood pressure, prothrombin time, D-Dimer, fibrinogen, NT-proBNP, left atrial diameter, left ventricular ejection fraction, and interventricular septal amplitude. For the Lp(a) group: Q1: Lp(a) ≤ 91.5 mg/L; Q2: 91.5 < Lp(a) ≤ 164 mg/L; Q3: 164 < Lp(a) ≤ 266 mg/L; Q4: Lp(a) > 266 mg/L. For the LAD group: Q1: LAD ≤ 31 mm; Q2: 31 < LAD ≤ 35 mm; Q3: 35 < LAD ≤ 39.5 mm; Q4: LAD > 39.5 mm

However, it is necessary to point out that there may be an imbalance in the baseline characteristics of the patients, which could potentially affect the results. Therefore, PSM analysis was used for validation. Based on the ideal cut-off value, the patients were split into a high Lp(a) group (≥ 224.5 mg/L) and a control group (< 224.5 mg/L). In the high Lp(a) subgroup, there were substantially more cases of LAT/SEC (36.1% vs. 9.6%, *P* < 0.001). After incorporating variables that showed statistical differences between the two groups and non-paroxysmal atrial fibrillation in the PSM analysis, the confounding variables were balanced (Table 4). The high Lp(a) subgroup continued to have a significantly greater incidence of LAT/SEC (35.8% vs. 13.9%,* P* < 0.001). Similarly, patients were split into those with an enlarged LAD (≥ 36.5 mm) and those without (< 36.5 mm) according to the ideal cut-off value (

**Table 4:** Comparison between the two groups before and after 1:1 propensity score matching

	Before matching			After matching		
	Lp(a) <224.5mg/L (*n*=323)	Lp(a)≥224.5mg/L (*n*=158)	*P*	Lp(a)<224.5mg/L (*n*=151)	Lp(a)≥224.5mg/L (*n*=151)	*P*
Paroxysmal AF, %	104(32.2)	55(34.8)	0.606	94(62.3)	100(66.2)	0.471
CHA_2_DS_2_-VASc			0.033*			0.815
M0F1	158(48.9)	61(38.6)		63(41.7)	61(40.4)	
M1F2	165(51.1)	97(61.4)		88(58.3)	90(59.6)	
Statins, %	76(23.5)	24(15.2)	0.034*	24(15.9)	24(15.9)	1.000
LVEF, %	67.0(62.0-70.0)	66.0(61.8-69.0)	0.047*	66.0(62.0-69.0)	66.0(62.0-69.0)	0.782
LAT/SEC, %	31(9.6)	57(36.1)	<0.001*	21(13.9)	54(35.8)	<0.001*

^*^P<0.05

*M0F1* 0 point in men or 1 point in women, *M1F2* 1 point in men or 2 points in women, *AF* Atrial fibrillation, *LAT/SEC* Left atrial thrombosis/left atrial spontaneous echo contrast, *Lp(a)* Lipoprotein(a), *LVEF* Left ventricular ejection fraction

Table 5). After conducting the chi-square test, the expanded LAD group had a considerably greater occurrence of LAT/SEC than in the control (27.1% vs. 6.4%, *P* < 0.001). After PSM, the results remained consistent (17.3% vs. 5.3%, *P* < 0.001). Therefore, the elevation of Lp(a) and LAD can both be considered risk markers for LAT/SEC.

**Table 5:** Comparison between the two groups before and after 1:1 propensity score matching

	Before matching			After matching		
	LAD<36.5mm (*n*=204)	LAD≥36.5mm (*n*=277)	*P*	LAD<36.5mm (*n*=75)	LAD≥36.5mm (*n*=75)	*P*
LAT/SEC, %	31(9.6)	57(36.1)	<0.001*	4(5.3)	13(17.3)	0.020*
CHA_2_DS_2_-VASc			0.025*			0.870
M0F1	105(51.5)	114(41.2)		35(46.7)	34(45.3)	
M1F2	99(48.5)	163(58.8)		40(53.3)	41(50.6)	
Paroxysmal AF, %	176(86.3)	146(52.7)	<0.001*	21(28.0)	21(28.0)	1.000
Age, years	54.50±9.95	58.51±7.54	<0.001*	54.50±9.95	58.51±7.54	0.840
BMI, kg/m^2^	24.66±2.74	25.58±3.17	0.001*	24.89±2.78	25.04±2.56	0.753
DBP, mmHg	79(72-86.75)	80(74-89.50)	0.044*	76(70-86)	79(73-88)	0.249
HR, bpm	73(65-82)	76(67-87)	0.019*	72(65-86)	74(67-84)	0.774
Heart failure, %	1(0.5)	9(3.2)	0.036*	1(1.3)	1(1.3)	1.000
Anticoagulants, %	51(25.0)	102(36.8)	0.006*	28(37.3)	22(29.3)	0.299
PLT, ×10^9^/L	200.20±60.48	189.13±53.38	0.034*	190.56±54.10	194.91±53.04	0.620
ALP, U/L	68(57-82)	72(59.50-87)	0.028*	72(59-88)	68(56-89)	0.665
γ-GT, U/L	19(14-30)	26(17-42)	<0.001*	18(14-31)	22(16-32)	0.116
Scr, umol/L	60.05±17.18	64.03±16.99	0.012*	63.23±17.51	64.56±15.86	0.626
TC, mmol/L	3.78(3.16-4.36)	3.61(3.04-4.13)	0.033*	3.74(3.23-4.15)	3.70(3.28-4.29)	0.703
TG, mmol/L	1.25(0.85-1.75)	1.06(0.82-1.49)	0.016*	1.14(0.86-1.60)	1.16(0.82-1.61)	0.901
LDL-c, mmol/L	2.28(1.73-2.77)	2.05(1.57-2.65)	0.021	2.31(1.81-2.61)	2.18(1.74-2.66)	0.991
PT, s	12.10(11.13-13.38)	13.05(11.60-14.48)	<0.001*	12.60(11.20-13.40)	12.50(11.30-14.00)	0.748
APTT, s	29.65(26.50-35.65)	31.25(27.10-37.10)	0.041*	30.50(26.40-36.40)	31.20(26.90-36.50)	0.913
NT-proBNP, pg/mL	101.45(43.36-247.85)	582.50(347.20-1064.00)	<0.001*	153.60(50.18-508.70)	388.50(180.50-627.80)	0.400
IVSA, mm	8.0(7.0-9.0)	8.0(7.0-9.0)	0.001*	8.0(7.0-9.0)	8.0(7.0-9.0)	0.954
MPAD, mm	21.0(20.0-23.0)	22.0(21.0-24.0)	<0.001*	21.0(20.0-23.0)	22.0(21.0-23.0)	0.271
LEVF, %	68.0(65.0-71.0)	64.0(59.0-69.0)	<0.001*	67.0(63.0-70.0)	66.0(63.0-70.0)	0.829

^*^P <0.05. AF, atrial fibrillation

*ALP* Alkaline phosphatase, *APTT* Activated partial thromboplastin time, *BMI* Body Mass Index, *DBP* Diastolic Blood Pressure, *HR* Heart rate, *IVSA* Interventricular septal amplitude, *LAD* Left atrial diameter, *LAT/SEC* Left atrial thrombosis/left atrial spontaneous echo contrast, *LVEF* Left ventricular ejection fraction, *LDL-C* Low-density lipoprotein cholesterol, *M0F1* 0 point in men or 1 point in women, *M1F2* 1 point in men or 2 points in women; *MPAD* Main pulmonary artery, *PLT* Platelet, *PT* Prothrombin time, *Scr* Serum creatinine, *TC* Total cholesterol, *TG* Triglyceride, *γ-GT* γ-glutamyl transferase

### Lp(a) and LAD enhance the prediction accuracy of the CHA_2_DS_2_-VASc score

**Fig. 3:**
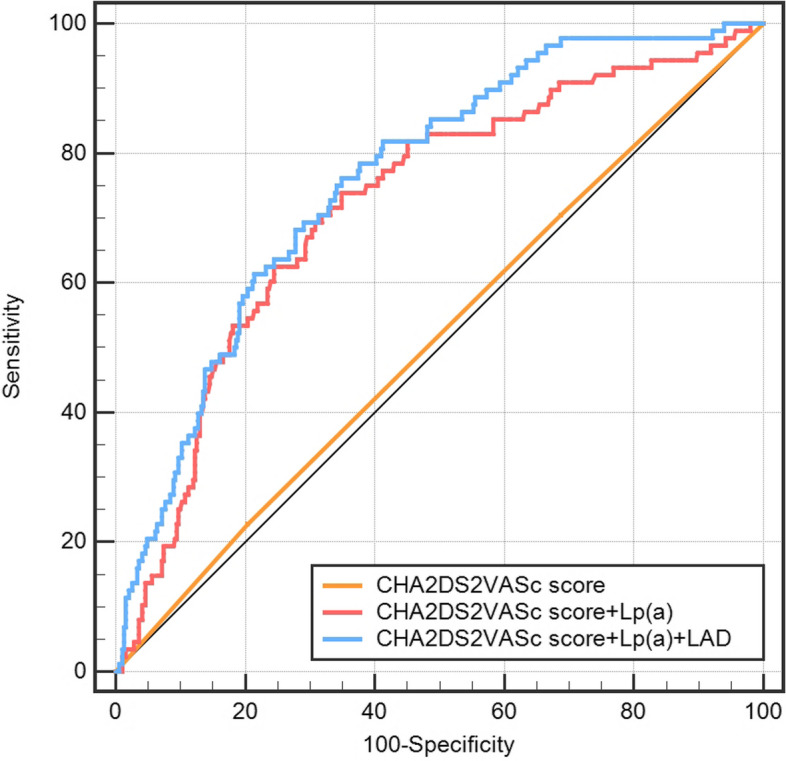
Receiver operating characteristic (ROC) analysis of each prediction model. Lp (a): lipoprotein (a); LAD: left atrial diameter

Given all this, enlarged LAD, increased Lp(a), and a history of CHD were predictors of LAT/SEC. Only LAD and Lp(a) were included in the new model because a history of CHD was already taken into account when calculating the CHA_2_DS_2_-VASc score. As shown in Table 6 and Fig. 3, the predictive ability for LAT/SEC of the CHA_2_DS_2_-VASc score was poor (AUC = 0.515, *P* > 0.05). After incorporating Lp(a) (Model A), the predictive value significantly improved (AUC difference = 0.206, Z = 4.848, *P* < 0.01). After combining Lp(a) and LAD (Model B), the value for LAT/SEC prediction was also improved (AUC difference = 0.245, Z = 6.259, *P* < 0.01). However, in comparison to Model A, Model B showed no statistically significant difference (AUC difference = 0.0394, Z = 1.595, *P* > 0.05).

**Table 6:** Comparison between the prediction models

	AUC	95%CI	*P*^†^	Z	*P*^‡^
CHA_2_DS_2_-VASc score	0.515	0.469-0.561	0.634	0.477	-
CHA_2_DS_2_-VASc score + Lp(a)	0.721	0.679-0.761	<0.01	4.848^*^	<0.01^*^
CHA_2_DS_2_-VASc score + Lp(a) +LAD	0.760	0.720-0.798	<0.01	6.259^*^	<0.01^*^
				1.595^**^	0.11^**^

^†^*P*-value for each ROC curve analysis

^‡^the *P*-value when comparing between the two models

*compared to the CHA_2_DS_2_-VASc score group

**compared to the CHA_2_DS_2_-VASc score + Lp (a) group

*AUC* Area under the receiver operating characteristic curve, *CI* Confidence interval, *LAD* Left atrial diameter, *Lp(a)* Lipoprotein (a)

## Discussion

The yearly increase in morbidity from atrial fibrillation (AF) has resulted in a greater burden of thrombotic events, particularly stroke, which is attributed to increased mortality [16]. LAT and SEC are considered to be directly associated with AF-induced thromboembolism [17], and oral anticoagulants are considered to be effective treatments for eliminating left atrial thrombosis [3]. Current guidelines advocate oral anticoagulants for patients classified as "high risk" according to their CHA_2_DS_2_-VASc score (men ≥ 2; women ≥ 3). However, in patients who have low CHA_2_DS_2_-VASc scores, there is a debate regarding the use of anticoagulants. Previous studies have shown that thrombotic events still occur in NVAF patients at "low risk". According to a study conducted in Denmark involving 39,400 NVAF patients, untreated low-risk patients had an annual incidence of ischemic stroke of 0.49% [18]. Similarly, another study conducted in Taiwan revealed that, throughout the median follow-up of five years, 14.4% of men and 14.9% of women with low CHA_2_DS_2_-VASc scores suffered an ischemic stroke, and the corresponding annual stroke rates were 2.75% and 2.55%, respectively [6]. Moreover, the CHA_2_DS_2_-VASc score primarily targets stroke as the primary outcome event rather than left atrial thrombosis. Notably, low-risk patients who had not experienced a prior stroke or embolic event were classified in the high-risk group only after an embolic event occurred. This finding suggested a delay in the prediction of the CHA_2_DS_2_-VASc score.

Current studies have reported different prevalence rates of LAT in NVAF patients, with an average of 9.8% [12]. This study revealed that LAT and SEC were present in 12.47% and 11.43%, respectively, of the total study population, which might be higher than the rates reported in previous research. The disparity in findings could be attributed to potential selection bias and referral bias within the research, as well as variations in the demographics of the study populations. However, more importantly, these findings indicate that the emergence of LAT/SEC may be influenced by factors other than the CHA_2_DS_2_-VASc score. Hence, this investigation aimed to identify NVAF patients categorized as "low risk" based on conventional stroke risk assessment, yet are at elevated risk for AF-related embolic events, and to institute prompt clinical interventions to mitigate the occurrence of such events.

Lp(a) has been recognized as a possible predictor of the development of atherosclerosis and thrombosis [19–21]. The findings of previous study imply that Lp(a)'s pro-thrombotic impact is predominantly mediated by its apolipoprotein(a) (ApoA) component, which has a very high resemblance to plasminogen [22]. Lp(a) might bind competitively to the fibrin surface, tissue-type plasminogen activator, and cell receptors, consequently reducing the generation of plasmin and ultimately impairing fibrinolytic effects [23]. Furthermore, Lp(a) attaches to heparin as well as to heparan sulfate, thereby neutralizing their thrombosis-inhibiting effects [24]. Additionally, Lp(a) has the ability to induce the release of plasminogen activator inhibitor-1, leading to unbalanced plasma fibrinolysis and coagulation systems, and ultimately promoting thrombosis [25]. Moreover, it has also been discovered that Lp(a) promotes thrombosis by attaching to and deactivating tissue factor pathway inhibitors (TFPI) [26]. Consequently, the presence of Lp(a) disrupts the equilibrium between the coagulation and fibrinolytic systems under normal physiological circumstances, thereby facilitating thrombosis.

Most related research suggests that genetic inheritance is the primary factor affecting Lp(a) levels and holds steady for the majority of a person's lifetime [27, 28]. Due to variations in Lp(a) gene polymorphisms among different ethnic groups [7], there are inconsistencies in the cut-off points for assessing cardiovascular risk according to various guidelines and consensuses [29, 30]. This is the first study demonstrating a substantial interaction between Lp(a) levels and the frequency of LAT/SEC among NVAF patients who have low CHA_2_DS_2_-VASc scores, with an optimal predicted cut-off value of 224.5 mg/L.

In this study, except for Lp(a), the LAD was also discovered to be a predictor of LAT/SEC. Left atrial enlargement is not only a consequence of atrial fibrillation but is also considered a predictor of thrombotic events [31–33]. According to a study of 705 NVAF patients, the occurrence of LAT/SEC was moderately predicted by enlargement of the left atrium [34]. Zhou M. et al. indicated that among NVAF patients with a low risk of stroke, an enlarged left atrium was a risk indicator for LAT [35]. A prospective community-based survey revealed that stroke rates were higher in NVAF patients with a LAD exceeding 45 mm, and LA enlargement has been demonstrated to be a substantial indicator of both extracranial embolism events and stroke [36].

The LAT/SEC group had a larger LAD than the non-LAT/SEC group did among the NVAF patients (39.0 (35.0-43.0) vs. 34.0 (31.0-39.0), *P* < 0.001). Additionally, for every 1 mm increase in LAD, the risk of LAT/SEC increased by 7.2%, indicating a positive correlation between LAD and LAT/SEC. The CHA_2_DS_2_-VASc score's ability to predict LAT/SEC was significantly improved by the addition of Lp(a) and LAD (AUC difference = 0.245, Z = 6.259, *P* < 0.01). Therefore, a plasma Lp(a) level ≥224.5 mg/L and a LAD ≥36.5 mm indicate a heightened likelihood of LAT formation, and TEE should be performed to detect intra-atrial thrombosis when assessing thrombosis risk.

### Study strengths and limitations

The risk factors for LAT/SEC among NVAF patients with low CHA_2_DS_2_-VASc scores have been the subject of only a small amount of research to date. This research is the first to identify the predictive utility of increased Lp(a) levels for LAT/SEC, with a threshold of 224.5 mg/L. Consistent with the findings of previous research, this study also found that left atrial dilatation is a predictor of LAT/SEC. The predictive capacity of the CHA_2_DS_2_-VASc score for LAT/SEC was significantly improved by incorporating Lp(a) and LAD. These findings suggest that when assessing stroke risk in NVAF patients, Lp(a) and LAD should be taken into account together with the CHA_2_DS_2_-VASc score. This comprehensive approach might help recognize patients who are at potential risk of thrombosis and enable the timely administration of anticoagulation therapy to improve the long-term prognosis.

However, there were several limitations that should be addressed. First, only individuals who underwent TEE examination during their hospitalization were included in this single-center study. This may have led to some selection bias. Second, the findings of this research could be influenced by the variability in referrals. The majority of patients treated at our facility were referred by subordinate hospitals, which may involve additional complexity and greater clinical complications. As a result, this could lead to a higher rate of positive outcomes. Notably, Lp(a) levels vary among different ethnicities. This study focused primarily on the Chinese Han population, so the findings may not be generalizable to other races. In addition, due to the relatively limited clinical data collected, this study used only the LAD to evaluate left atrial enlargement. Other indicators, such as left atrial appendage morphology and left atrium volume index, were not included in the systematic evaluation of left atrium function. Furthermore, a prospective study may be needed to verify the conclusions of this study due to its retrospective nature.

## Conclusion

Elevated Lp(a) and enlarged LAD were independent risk factors for LAT/SEC among NVAF patients with low CHA_2_DS_2_-VASc scores. The prediction accuracy of the CHA_2_DS_2_-VASc score for LAT/SEC was significantly improved by the addition of Lp(a) and LAD. When evaluating the stroke risk in patients with NVAF, Lp(a) and LAD should be taken into account together with the CHA_2_DS_2_-VASc score.

## Data Availability

The datasets used and/or analyzed during the current study are available from the corresponding author upon reasonable request.

## Abbreviations

AF: Atrial Fibrillation
ApoA: Apolipoprotein(a)
APTT: Activated Partial Thrombin Time
AUC: Area Under the Curve
CHD: Coronary Heart Disease
CI: Confidence Interval
IVSA: Interventricular Septal Amplitude
LA: Left Atrium
LAAC: Left Atrial Appendage Closure
LAD: Left Atrial Diameter
LAT: Left Atrial Thrombosis
Lp(a): Lipoprotein(a)
LVEF: Left Ventricular Ejection Fraction
NVAF: Non-valvular Atrial Fibrillation
OR: Odds Ratio
PSM: Propensity Score Matching
PT: Prothrombin Time
RFCA: Radio-Frequency Catheter Ablation
ROC: Receiver Operating Characteristic
SEC: Spontaneous Echo Contrast
TEE: Transesophageal Echocardiography
TFPI: Tissue Factor Pathway Inhibitors
TIA: Transient Ischemic Attack
TTE: Transthoracic Echocardiography
